# [^123^I]CC1: A PARP-Targeting, Auger Electron–Emitting Radiopharmaceutical for Radionuclide Therapy of Cancer

**DOI:** 10.2967/jnumed.123.265429

**Published:** 2023-12

**Authors:** Chung Ying Chan, Zijun Chen, Florian Guibbal, Gemma Dias, Gianluca Destro, Edward O’Neill, Mathew Veal, Doreen Lau, Michael Mosley, Thomas C. Wilson, Véronique Gouverneur, Bart Cornelissen

**Affiliations:** 1 MRC Oxford Institute for Radiation Oncology, Department of Oncology, University of Oxford, Oxford, United Kingdom;; 2 Chemistry Research Laboratory, Department of Chemistry, University of Oxford, Oxford, United Kingdom; and; 3 Department of Nuclear Medicine and Molecular Imaging, University Medical Center Groningen, University of Groningen, Groningen, The Netherlands

**Keywords:** PARP, radionuclide therapy, radiopharmaceuticals, ^123^I, Auger

## Abstract

Poly(adenosine diphosphate ribose) polymerase (PARP) has emerged as an effective therapeutic strategy against cancer that targets the DNA damage repair enzyme. PARP-targeting compounds radiolabeled with an Auger electron–emitting radionuclide can be trapped close to damaged DNA in tumor tissue, where high ionizing potential and short range lead Auger electrons to kill cancer cells through the creation of complex DNA damage, with minimal damage to surrounding normal tissue. Here, we report on [^123^I]CC1, an ^123^I-labeled PARP inhibitor for radioligand therapy of cancer. **Methods:** Copper-mediated ^123^I iododeboronation of a boronic pinacol ester precursor afforded [^123^I]CC1. The level and specificity of cell uptake and the therapeutic efficacy of [^123^I]CC1 were determined in human breast carcinoma, pancreatic adenocarcinoma, and glioblastoma cells. Tumor uptake and tumor growth inhibition of [^123^I]CC1 were assessed in mice bearing human cancer xenografts (MDA-MB-231, PSN1, and U87MG). **Results:** In vitro and in vivo studies showed selective uptake of [^123^I]CC1 in all models. Significantly reduced clonogenicity, a proxy for tumor growth inhibition by ionizing radiation in vivo, was observed in vitro after treatment with as little as 10 Bq [^123^I]CC1. Biodistribution at 1 h after intravenous administration showed PSN1 tumor xenograft uptake of 0.9 ± 0.06 percentage injected dose per gram of tissue. Intravenous administration of a relatively low amount of [^123^I]CC1 (3 MBq) was able to significantly inhibit PSN1 xenograft tumor growth but was less effective in xenografts that expressed less PARP. [^123^I]CC1 did not cause significant toxicity to normal tissues. **Conclusion:** Taken together, these results show the potential of [^123^I]CC1 as a radioligand therapy for PARP-expressing cancers.

## Visual Abstract

**Figure:**
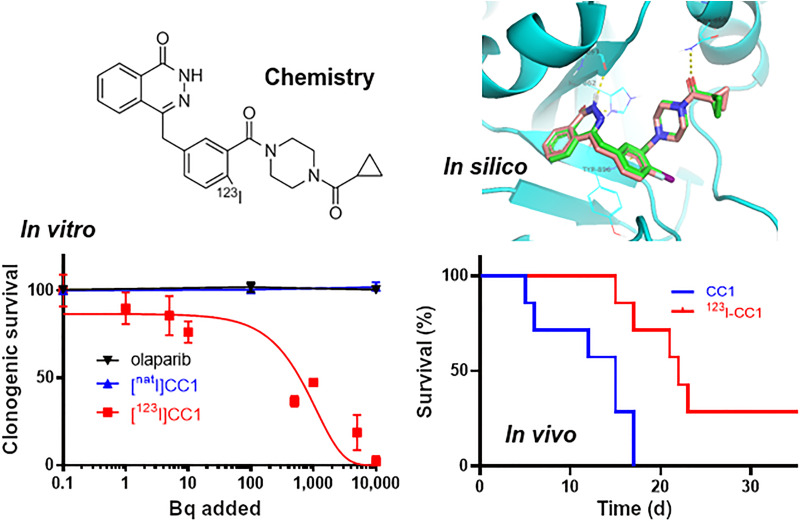

Poly(adenosine diphosphate ribose) polymerase (PARP) inhibitors function as competitive inhibitors of the NAD^+^ binding pocket of PARP enzymes, a class of DNA damage repair enzymes. They inhibit the catalytic function of PARP1 (often called PARP), PARP2, and PARP3, as well as other members of that family of enzymes (1). Of these, PARP1 is the most abundant and is a critical enzyme for the repair of single-strand DNA damage. PARP inhibitors prevent polyadenoribosylation of target proteins, also stopping autopolyadenoribosylation and thus preventing disengagement from the enzyme from the broken DNA, thereby trapping it (2).

PARP1 expression in tumor tissue tends to be severalfold higher than in surrounding tissue (3), and PARP inhibitors get trapped close to DNA, making them vehicles for transporting therapeutic radionuclides with the aim of delivering ionizing radiation to tumor DNA. This would cause DNA damage and tumor cell death. Auger electron–emitting radionuclides are particularly suited for this approach (4). Auger electrons are short-range emissions from the electron cloud of decaying radionuclides. Auger electrons possess little kinetic energy and therefore travel a short range of only a few nanometers. However, their tendency to be released in cascades causes all their ionizing energy to be absorbed in a small space. When the radionuclide is delivered close to DNA, the Auger electron emissions are densely ionizing and cause complex, hard-to-repair DNA damage (4*,*^5^).

In recent years, radiolabeled PARP inhibitors have been developed for molecular imaging and radionuclide therapy. An overview, including radiolabeled versions of the PARP inhibitors olaparib (6*,*^7^), rucaparib (8*,*^9^), and talazoparib (10*,*^11^), is given in several review articles (1*,*^12^*,*^13^). Two radiolabeled compounds are furthest along the translational pipeline, with clinical trials under way: [^18^F]fluorthanatrace (14–17) and [^18^F]PARPi (6*,*^18^–^20^). Radionuclide therapy targeting PARP with α-, β-, and Auger electron emitters has been described using ^123^I-, ^125^I-, ^131^I-, ^125^I-, ^77^Br-, or ^211^At-labeled compounds that resemble olaparib- or rucaparib-like structures (9*,*^21^–^27^).

Previously, our group described a radioisotopolog of the PARP inhibitor olaparib, [^18^F]olaparib (7), which we were able to radiofluorinate by copper-assisted fluorodeboronation of a boronic pinacol ester precursor. Here, we show that the radioiodinated analog [^123^I]CC1, an Auger electron–emitting therapeutic radiopharmaceutical that targets PARP, is exquisitely radiotoxic to PARP-expressing tumor cells and causes marked tumor growth inhibition without causing gross toxicity.

## MATERIALS AND METHODS

### General

**FIGURE 1.**
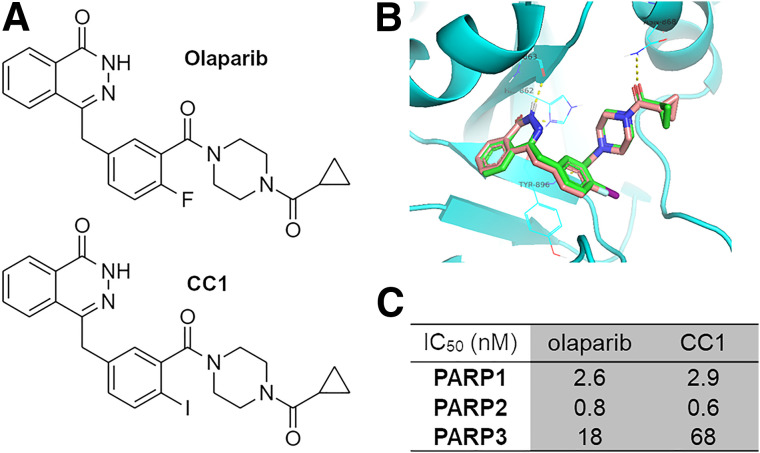
(A) Chemical structures of olaparib and CC1. (B) Molecular docking of olaparib (brown backbone) and CC1 (green) to PARP1 shows excellent overlap. (C) Cell-free enzymatic inhibition of PARP1, PARP2, and PARP3 by CC1 or olaparib. IC_50_ = inhibitory concentration of 50%.

The synthesis of CC1 (Fig. 1) was adapted from Wilson et al. (7). After preparative chromatography, CC1 was obtained with an overall yield of 9% (chemical purity > 95%) over 7 steps from commercially available compounds (Supplemental Fig. 1 [supplemental materials are available at http://jnm.snmjournals.org]) (7).

A commercially available assay (catalog number 4671-096-K; Trevigen) was used to measure PARP1, PARP2, and PARP3 catalytic activities in vitro, in a cell-free assay, and in the presence of varying concentrations of established PARP inhibitors and CC1, according to the manufacturer’s instructions. Elacridar was used as a negative control.

### Synthesis of [^123^I]CC1

**FIGURE 2.**
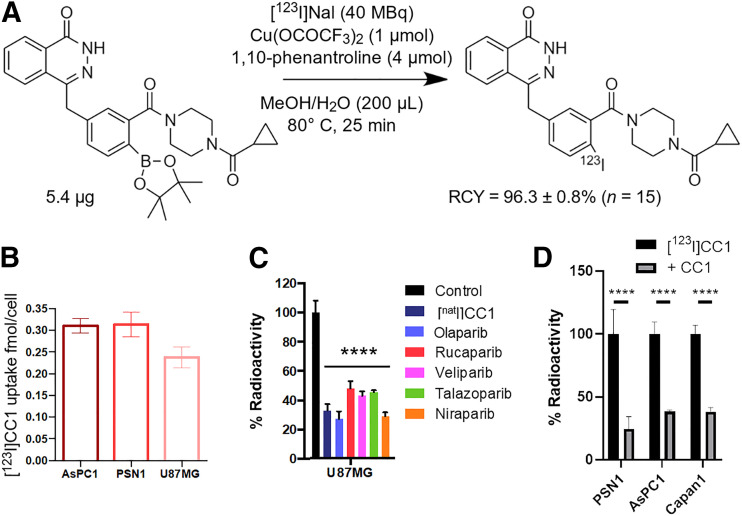
(A) Radiosynthesis of [^123^I]CC1 by copper-assisted iododeboronation. (B) Uptake of [^123^I]CC1 in AsPC1, PSN1, and U87MG cells after 1 h of exposure. (C) Blocking of [^123^I]CC1 uptake in U87MG cells by panel of unlabeled CC1 or other PARP inhibitors. (D) Uptake of [^123^I]CC1 in PSN1, AsPC1, or Capan1 cells. ****P < 0.0001. RCY = Radiochemical yield.

A boronic pinacol ester was synthesized as precursor **9.** The supplemental materials give a full description of the synthetic methodology. Sodium [^123^I]iodide was provided in 0.05 M NaOH (GE Healthcare). [^123^I]CC1 was synthesized from precursor **9** via a copper-mediated iododeboronation reaction (Fig. 2), using a procedure modified from our previous work (7). The supplemental materials give a full description of the methodology (Supplemental Figs. 1–4). Non–decay-corrected radiochemical yields of more than 95% and a molar activity (A_m_) range of 18–342 GBq/μmol were obtained. Radiochemical yield was greater than 95% (non–decay-corrected), over a 2-h synthesis.

### Cell Culture

Human malignant glioma (U87MG) cells were donated by Professor Nicola Sibson at our institute and maintained in high-glucose Dulbecco modified Eagle medium supplemented with 10% fetal bovine serum (Gibco), 2 mM l-glutamine, 100 units/mL penicillin, and 0.1 mg/mL streptomycin (Gibco). Pancreatic adenocarcinoma cells (AsPC1, PSN1, and Capan1) and breast cancer cells (MDA-MB-231) were purchased from the American Type Culture Collection and maintained in RPMI medium supplemented with 10% fetal bovine serum (Gibco), 2 mM l-glutamine, 100 units/mL penicillin, and 0.1 mg/mL streptomycin (Gibco). Cells were grown under a humidified environment at 37°C and 5% CO_2_. Cells were harvested and passaged using trypsin–ethylenediaminetetraacetic acid (EDTA) solution. Cells were used for no more than 20 passages after resuscitation from liquid nitrogen storage. All cells were authenticated by the provider and short tandem repeat profiling and were tested regularly for the absence of *Mycoplasma.*

Relative expression of PARP1, PARP2, and PARP3 was determined by flow cytometry of live cells. Full details are available in the supplemental materials.

### In Vitro Uptake and Specificity of [^123^I]CC1

AsPC1 cells (1 × 10^5^ cells per well), PSN1 or Capan1 cells (7.5 × 10^4^ cells per well), or U87MG cells (1 × 10^5^ cells per well) were prepared using trypsin-EDTA, seeded separately in 24-well plates containing growth medium, and allowed to adhere for at least 20 h. Cells were washed and exposed to unlabeled PARP inhibitors and unlabeled CC1 (0 or 100 μM in 270 μL of growth medium) for 45 min at 37°C. Then, [^123^I]CC1 (30 μL, 11–100 kBq, 18–255 GBq/μmol; overall CC1, 0.6–3.7 pmol) was added, and the cells were incubated at 37°C for 45 min. The cell culture medium was removed, and the cells were washed with phosphate-buffered saline (PBS). Cells were lysed using RIPA buffer (950 mM Tris, pH 8.0; 1% NP40; 0.5% sodium deoxycholate; 0.1% sodium dodecyl sulfate; and 150 mM sodium chloride) for 15 min at room temperature, and the amount of ^123^I in cell lysates was measured using an automated γ-counter (PerkinElmer). Before the [^123^I]CC1 treatment, cells were counted using an automated cell counter.

In a separate experiment, cells were prepared in a similar manner but were washed and exposed to [^123^I]CC1 (39–50 kBq, 24.8–138.8 GBq/μmol; overall CC1, 0.3–2 pmol) at 37°C for different intervals (1–120 min). The amount of ^123^I in cell lysates was measured as described earlier. Separately, cells were exposed to [^123^I]CC1 for 30 min at 37°C, washed with PBS, and supplied with fresh growth medium. Then, the amount of ^123^I associated with cells was measured after varying intervals as described earlier.

### Colony Formation Assay

After harvesting using Accutase (Innovative Cell Technologies, Inc.), aliquots of 10,000 cells (PSN1 or U87MG), in 0.2 mL of cell growth medium in 0.5-mL Eppendorf tubes, were exposed to increasing amounts of [^123^I]CC1 (0–10 kBq, 18 GBq/μmol; total CC1, 0–0.55 pmol) or equivalent concentrations of unlabeled olaparib or CC1 (0–0.55 pmol) for 60 min at 37°C. After incubation, cell suspensions were diluted to 3 mL of growth medium, with a fraction of the cells (1.5 mL, 5,000 cells) seeded in duplicate in 6-well plates before medium was added to bring the total to 3 mL. Two weeks later, the number of colonies (>50 cells) was measured after washing and staining using crystal violet (1 mg/L in a 1:1 water-to-methanol ratio) (28).

### Quantification of Nuclear Protein Expression After [^123^I]CC1 Treatment

PSN1 or U87MG (1 × 10^6^ cells per well in 2 mL of growth medium) was seeded in 6-well plates and allowed to adhere overnight. Cells were washed and exposed to [^123^I]CC1 (30 μL, 50 kBq, 18 GBq/μmol, in 2 mL of growth medium) at 37°C for 1 h. After washing, cells were supplied with fresh growth medium for another 1 or 24 h. Cells were harvested using trypsin-EDTA solution, washed with fluorescence-activated cell sorting buffer (PBS, 2% fetal bovine serum, 1 mM EDTA, and 0.1% NaN_3_), and centrifuged at 500*g* for 5 min. Relative expression of PARP1 and PARP2 was measured using flow cytometry (supplemental materials). γH2AX expression, as a measure of DNA double-strand-break damage, was assessed in a similar manner.

### SPECT/CT Imaging and Biodistribution of [^123^I]CC1 in Immunocompromised Mice Bearing Xenografts

All animal procedures were performed in accordance with the U.K. Home Office’s Guidance on the Operation of Animals (Scientific Procedures) Act of 1986 and the Animal Research: Reporting of In Vivo Experiments guidelines. Local ethical committee approval was obtained (PPL PA1B5C52F, University of Oxford). Female immunocompromised BALB/c *nu/nu* (OlaHsd-*Foxn1^nu^*) mice, aged 4–6 wk, were purchased from Envigo. Animals were housed in individually ventilated cages, up to 6 mice per cage, in an artificial day–night cycle facility. Food and water were provided ad libitum. PSN1, U87MG, or MDA-MB-231 cells were harvested using trypsin-EDTA, washed twice with PBS, and reconstituted in PBS:Matrigel Matrix High Concentration (Corning) (1:1). Cell suspensions containing 10^6^ cells were injected subcutaneously into the hind right flank and allowed to form tumors. Tumor sizes were determined by caliper.

When tumors reached 50–200 mm^3^ (after ∼4–6 wk), animals were administered [^123^I]CC1 (3 MBq in 100 μL of PBS; A_m_, 26.1–124.3 GBq/μmol) by intravenous bolus injection via the lateral tail vein. To evaluate the specificity of tumor uptake, excess unlabeled, and structurally unrelated, rucaparib (0.5 mg) was coadministered as a blocking agent in some animals. Then, 1, 2, or 24 h after radiolabeled compound injection, animals were euthanized (*n* = 3 per group). Selected organs, tissues, and blood were removed, and the percentage injected activity per gram of tissue (%IA/g) was determined using a Hidex automated γ-counter.

A selected number of animals (*n* = 3) were anesthetized using 2% isoflurane, and dynamic SPECT/CT imaging was performed over 1 h using a MILabs VECTor4 camera equipped with an ultra-high-resolution rat or mouse collimator (1.8 mm), followed by a cone-beam CT scan (55 kV, 0.19 mA) for anatomic reference and attenuation correction. Anesthesia was maintained using isoflurane throughout the duration of image acquisition. SPECT images were reconstructed using U-SPECT-Rec3.22 software (MILabs), which applied a pixel-based algorithm, ordered-subset expectation maximization, with 4 subsets, 4 iterations, and a 0.6-mm voxel size for ^123^I (energy window settings, 141.3–172.7 keV). Reconstructed SPECT and CT images were viewed and analyzed using PMOD version 3.37 (PMOD Technologies). Localization of [^123^I]CC1 in PSN1 xenografts was determined ex vivo using autoradiography performed on frozen tumor sections (10 μm; Cyclone; Perkin Elmer).

### Toxicology

Hematoxylin and eosin staining and γH2AX staining were performed on selected tissues at 24 h and 28 d after intravenous administration of 3 MBq [^123^I]CC1 to otherwise naïve C57BL/6 mice (*n* = 3 per time point). Liver, spleen, kidneys, and intestines were harvested and immediately washed and fixed in 10% formalin for 24 h. Tissues were sectioned and stained for hematoxylin and eosin and for γH2AX. Stained tissue sections were investigated blindly by an experienced veterinary pathologist and compared with age-matched, nontreated control animals.

### In Vivo Therapy Studies

Mice bearing PSN1 xenografts, with average tumor sizes of 50–200 mm^3^, were randomly grouped into cohorts and intravenously injected with [^123^I]CC1 (3 MBq in 100 μL of PBS; A_m_, 120–340 GBq/μmol) or an equivalent amount of unlabeled CC1 (*n* = 7 per group). Mice were monitored daily. Study endpoints were humane endpoints, including a tumor size of more than 1,000 mm^3^ or weight loss of more than 15%. The procedure was repeated in mice bearing U87MG xenografts (*n* = 9 per group) or MDA-MB-231 xenografts (*n* = 3 per group).

### Dosimetry

In vitro, the absorbed radiation dose to the nucleus was determined by MIRDcell package version 3.10 (29–31), using uptake and retention data in U87MG cells. Cell and cell nuclear dimensions were approximated as concentric circles of sizes as determined by confocal microscopy, assuming concentric circular geometry (a 14-μm cell diameter and an 8-μm nucleus diameter). Cross-dose was assessed but found to be insignificant. The entire [^123^I]CC1 was assumed to be contained in the nucleus of the cell. Methodology for dosimetry was applied as in Pirovano et al. (29).

### Statistical Analysis

All data were obtained at least in triplicate. All statistical analyses and nonlinear regressions were performed using GraphPad Prism version 8 or higher (GraphPad Software). Data were tested for normality and analyzed as appropriate by 1- or 2-way ANOVA. Results are reported as mean ± SD, unless stated otherwise.

## RESULTS

### CC1 Is a Selective and Potent PARP Inhibitor

CC1, similar in structure to olaparib (Fig. 1A), fits well within the NAD^+^ binding pocket of PARP1, similar to the fit of olaparib (Fig. 1B). CC1 proved to be a potent PARP inhibitor, with cell-free values for an inhibitory concentration of 50%, determined in-house, of 2.9 and 0.6 nM for PARP1 and PARP2, respectively (Fig. 1C; Supplemental Fig. 5). These were comparable to the values for olaparib (2.6 and 0.8 nM in the same assay). In contrast, PARP3 inhibition by CC1 was less pronounced (68 nM) than it was by olaparib (18 nM). Separately, 10 nM CC1 was able to inhibit PARP1, PARP2, and to a lesser extent, PARP6 (similar to inhibition by olaparib; results not shown). If interpreted as a proxy for PARP binding, CC1 binds to PARP1 and PARP2 but less so to PARP3.

### [^123^I]CC1 Uptake in Cells In Vitro Is PARP-Selective

[^123^I]CC1 was produced reliably, in good radiochemical yield, and with high A_m_ (Fig. 2A). AsPC1, PSN1, and U87MG cells expressed PARP1 and PARP2 to varying degrees; PSN1 had the highest PARP1 expression, followed by AsPC1 and then U87MG (Supplemental Fig. 6). [^123^I]CC1 was taken up in all 3 cell lines within minutes, plateauing after 1 h (Supplemental Fig. 7A). [^123^I]CC1 was retained briefly in cells (Supplemental Fig. 7B). [^123^I]CC1 was taken up selectively in AsPC1, PSN1, and U87MG cells, with uptake of around 0.3 fmol/cell (out of 0.1 nM [^123^I]CC1 added; Figs. 2B–2D). The addition of structurally related or unrelated nonlabeled PARP inhibitors significantly reduced the cell-associated amount of [^123^I]CC1 in all cell lines (*P* < 0.0001), suggesting PARP-selective binding of [^123^I]CC1 (Fig. 2D).

### [^123^I]CC1 Causes DNA Damage and Reduces Clonogenic Survival

**FIGURE 3.**
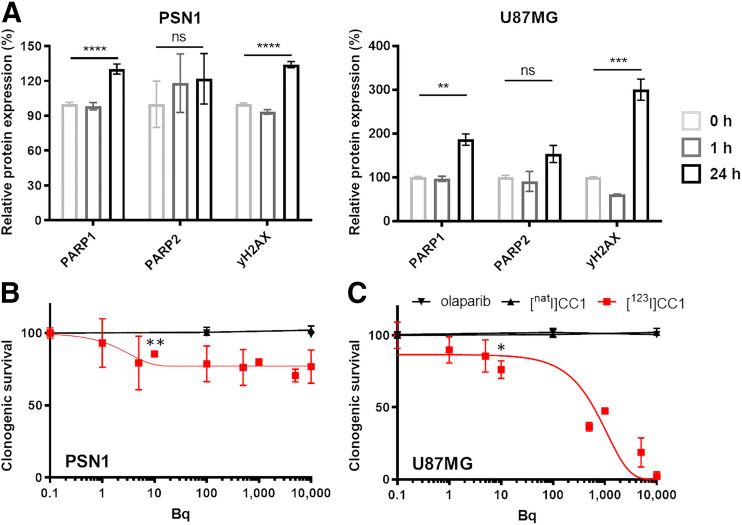
(A) Flow cytometry measurements of PARP1, PARP2, and γH2AX expression at 1 or 24 h after 1 h of exposure to [^123^I]CC1 (50 kBq, 138.8 GBq/μmol, in 2 mL of growth medium) in PSN1 and U87MG cells, relative to 0 h. (B and C) Clonogenic survival of PSN1 or U87MG cells, comparing exposure of cells for 1 h to [^123^I]CC1 (0–10 kBq in 0.2 mL of growth medium), nonlabeled CC1, or olaparib. ns, *P* > 0.05. *P < 0.05. **P < 0.01. ***P < 0.001. ****P < 0.0001. ns = not significant.

γH2AX expression, a marker of DNA double-strand breaks, increased markedly 24 h after a 1-h exposure of PSN1 and U87MG cells to a small amount of [^123^I]CC1 (50 kBq; *P* < 0.001; Fig. 3A). Exposure of both cell types to [^123^I]CC1 resulted in increased expression of PARP1 at 24 h after a 1-h exposure (*P* < 0.01; Fig. 3A). In vitro cell dosimetry, calculated by MIRDcell using uptake and retention values (Supplemental Fig. 7), estimated the absorbed radiation dose (over 24 h) to be approximately 8 Gy.

Clonogenic survival of cells was significantly reduced by exposure to [^123^I]CC1 from added activities as small as 10 Bq (20 MBq/μmol; *P* = 0.02) for U87MG cells. Values for an inhibitory concentration of 50% for [^123^I]CC1 equated to 631 ± 35 Bq (in 200 μL of growth medium) for U87MG cells. Efficacy in PSN1 cells in vitro was less pronounced yet significant (*P* < 0.001). Equivalent amounts of CC1 or olaparib had no effect on clonogenic survival (Figs. 2B and 2C).

### Biodistribution of [^123^I]CC1 Shows a Hepatobiliary Clearance Pattern

**FIGURE 4.**
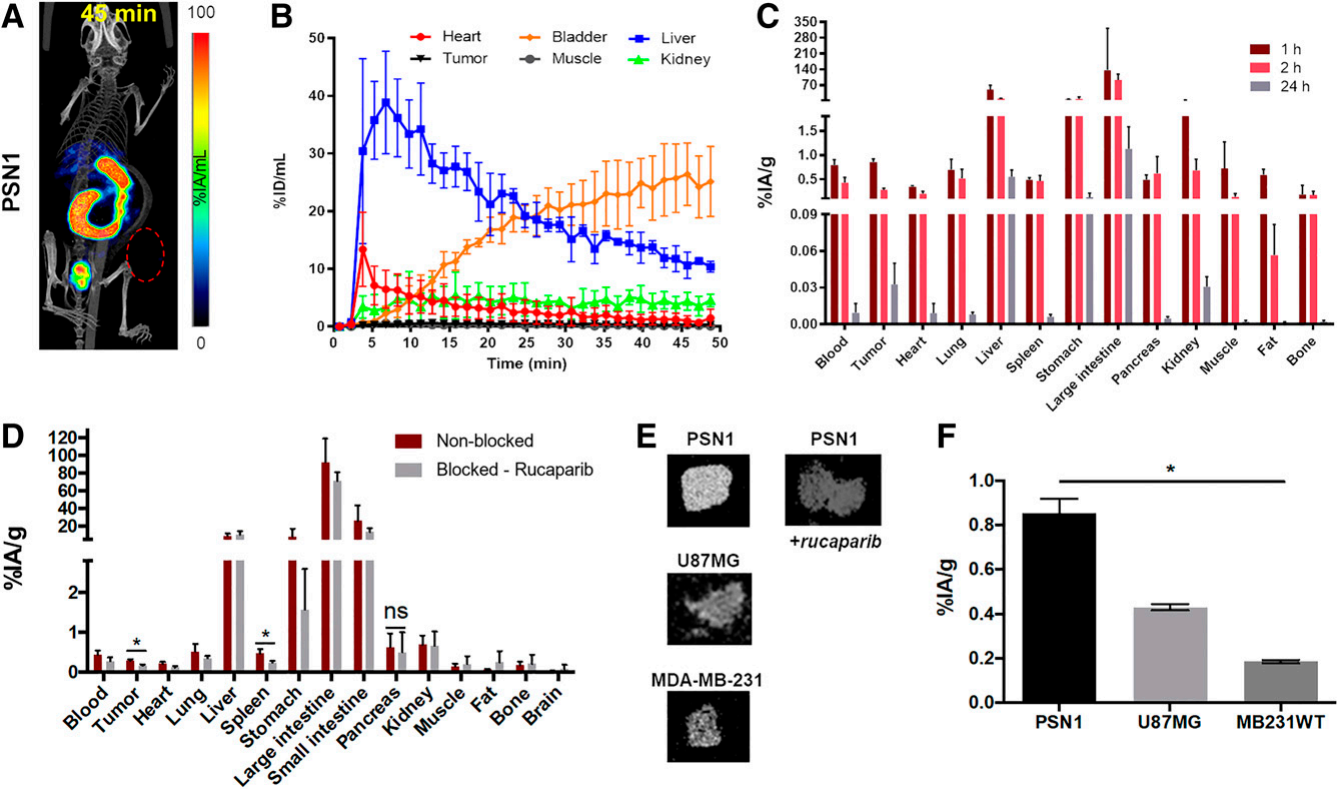
(A) SPECT image, 45 min after intravenous administration of [^123^I]CC1 (3 MBq; coronal maximum intensity projection). (B) Volume-of-interest analysis of dynamic SPECT images in selected tissues of mice bearing PSN1 xenografts. (C) Biodistribution of [^123^I]CC1 in selected tissues of mice bearing PSN1 xenografts at 1, 2, or 24 h after intravenous administration of [^123^I]CC1 (3 MBq). (D) Biodistribution in selected tissues of mice bearing PSN1 xenografts at 2 h after intravenous injection of [^123^I]CC1 (3 MBq) with or without excess unlabeled rucaparib (0.5 mg). (E) Autoradiography of tumor sections harvested 2 h after intravenous injection of [^123^I]CC1 (3 MBq) in mice bearing PSN1, U87MG, or MDA-MB-231 xenografts. (F) Tumor uptake 1 h after intravenous injection of [^123^I]CC1 (3 MBq) in mice bearing PSN1, U87MG, or MDA-MB-231 xenografts. ns, *P* > 0.05. *P < 0.05. %ID = percentage injected dose; ns = not significant.

Dynamic SPECT/CT imaging and biodistribution of [^123^I]CC1 (3 MBq, 20 GBq/μmol) were investigated in mice bearing PSN1 xenografts (Fig. 4; Supplemental Fig. 8). High uptake in liver and intestines indicated a hepatobiliary clearance pattern similar to that of other radiolabeled PARP inhibitors (Figs. 4A and 4B) (6–11). Blood clearance showed 2-phase decay with fast and slow half-lives of 16.85 and 1.35 min, resulting in a weighed half-life of 1.86 min (95% CI, 1.64–2.09 min, using an artificial data point at *t* = 0 and a blood volume of 2 mL; Fig. 4B). Cut-and-count biodistribution studies showed tumor uptake in PSN1 xenografts amounted to 0.9 ± 0.06 %IA/g at 1 h after intravenous administration, and normal-tissue uptake was in line with dynamic SPECT imaging (Fig. 4C). Studies showed 0.033 ± 0.017 %IA/g [^123^I]CC1 remained in the tumor 24 h after injection. Coadministration of excess rucaparib, to block the NAD^+^ binding pocket in the PARP enzymes, significantly reduced tumor uptake (*P* < 0.05; Fig. 4D), which was confirmed by autoradiography (Fig. 4E). Tumor uptake in U87MG and MDA-MB-231 xenografts was significantly lower than in PSN1 xenografts (0.46 ± 0.01 %IA/g for U87MG and 0.19 ± 0.01 %IA/g for MDA-MB-231 xenografts; Figs. 4E and 4F).

### [^123^I]CC1 Displays Limited Normal-Tissue Toxicity

**FIGURE 5.**
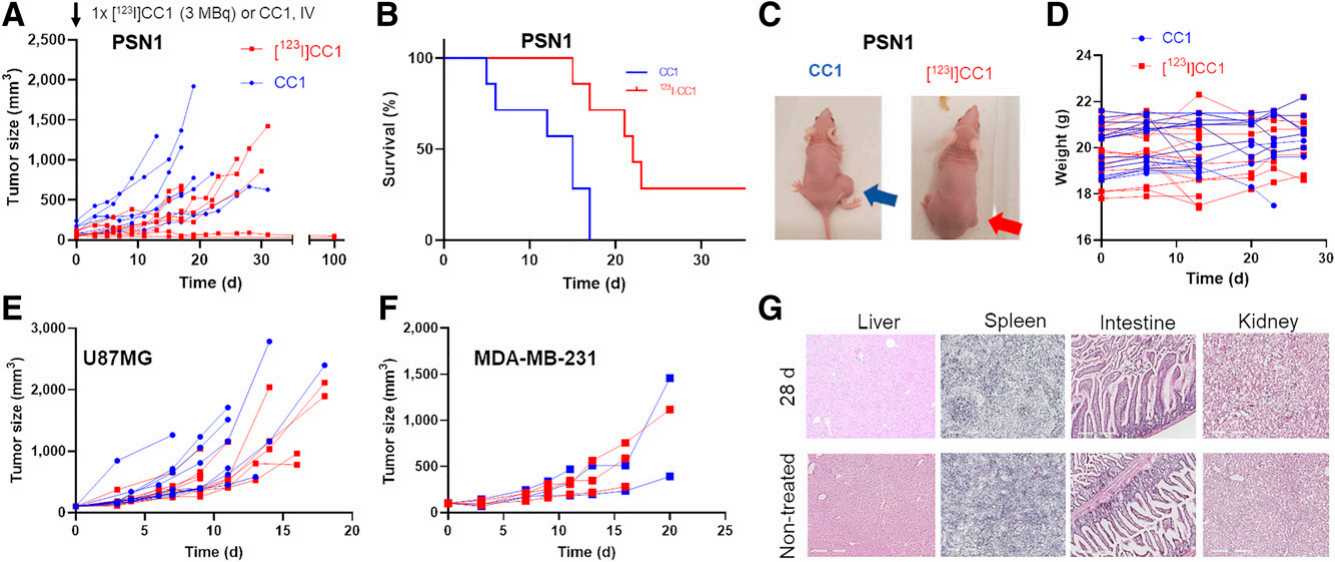
(A) Tumor growth after single intravenous administration of [^123^I]CC1 (3 MBq, 124.2–341.9 GBq/μmol) or equivalent amount of nonlabeled CC1 in mice bearing PSN1 xenografts. (B) Kaplan–Meier survival plots of A (survival event, V = 600 mm^3^). (C) Representative photographs of mice bearing PSN1 xenografts, 15 d after [^123^I]CC1 or CC1 administration. (D) Animal weights after administration of [^123^I]CC1 or CC1 (mixed tumor models). (E and F) Tumor growth curves after single intravenous administration of [^123^I]CC1 (3 MBq) or equivalent amount of nonlabeled CC1 in mice bearing U87MG or MDA-MB-231 xenografts. (G) Representative hematoxylin and eosin staining in selected normal tissues at 28 d after administration of [^123^I]CC1 (3 MBq) or untreated C57BL/6 mice. IV = intravenous.

With a view to use [^123^I]CC1 for radionuclide therapy of tumors, we evaluated whether the radiolabeled compound induced toxicity in normal tissue. Radiation-induced damage from exposure to [^123^I]CC1 may be expected in the liver and intestines because of its biodistribution pattern. We also evaluated the kidneys because of their partial renal clearance. In addition, because of the ability of the radiolabeled PARP inhibitor [^18^F]olaparib to bind specifically to splenic tissue (7), we looked at the spleen (Fig. 5).

No increase in γH2AX staining could be observed in the intestines, kidneys, liver, or spleen at either 24 h or 28 d after administration of [^123^I]CC1 (Supplemental Fig. 9). The intestines of mice administered [^123^I]CC1 (3 MBq) intravenously showed minimal proprial infiltration by lymphocytes and plasma cells. Scattered intact eosinophils were present within the propria, but no mice showed signs of generation or necrosis in the enterocytes, with apical brush borders remaining intact. Mitotic figures were regularly present and within normal counts. Observations were no different at 24 h or 28 d after administration. The kidneys showed no observable changes, whereas in the spleen, mild to moderate numbers of hemosiderophages were observed at 24 h and at 28 d after administration. No signs of necrosis were seen. In the liver, hepatocellular nuclei were centrally located and showed no signs of necrosis. Some small foci of extramedullary hematopoiesis were present. Mild anisocytosis and anisokaryosis were observed. A few individual scattered hepatocytes (0.1–0.2 per field) showed a shrunken shape, hypereosinophilic cytoplasm, and a shrunken nucleus with condensed chromatin, interpreted as pyknosis. The sole marked effect in the liver consisted of diffuse cytoplasmic pallor or rarefaction, created by optically empty feathery spaces and vacuoles and some remaining floccular granulated cytoplasmic material, often peripheralized. Effects were slightly more pronounced in animals 28 d after administration of [^123^I]CC1, although the difference was not statistically significant (*P* > 0.05).

### Potent Tumor Growth Inhibition by [^123^I]CC1 Occurs in Mice Bearing Pancreatic Ductal Adenocarcinoma Xenograft Tumors

Intravenous administration of relatively small amounts of [^123^I]CC1 (3 MBq) showed significant tumor growth delay in mice bearing PSN1 xenografts (Fig. 5). A single intravenous administration of [^123^I]CC1 led to significant inhibition of tumor growth compared with animals exposed to unlabeled CC1 (*P* = 0.04). This was not associated with signs of gross toxicity, as determined by a lack of weight loss of the mice (*P* > 0.05). We observed no effect resulting from the size of the tumor at time of administration (*P* > 0.05). U87MG xenografts responded less than PSN1 xenografts (*P* > 0.05), whereas MDA-MB-231 xenografts were not affected in their growth by the same amount of [^123^I]CC1 (*P* > 0.05).

## DISCUSSION

Radiolabeled PARP inhibitors for imaging with PET and SPECT have been used to visualize the pharmacokinetics of these drugs, quantify PARP inhibitor target occupancy, or visualize the effects of genotoxic therapies, such as radiation therapy and radioligand therapy, reviewed previously (32). In cancer patients, PET imaging using radiolabeled PARP inhibitors has been demonstrated to highlight PARP-expressing head-and-neck tumors (20), breast cancer (16*,*^33^), and ovarian cancer (34) and to predict the efficacy of PARP inhibitor treatment (35).

In addition, PARP-mediated uptake of PARP inhibitors labeled with β-, α-, or Auger electron–emitting radionuclides can be used for radioligand therapy of PARP-expressing tumors. In a series of preclinical studies, it has been shown that ^123^I-, ^125^I-, ^131^I-, ^77^Br-, or ^211^At-labeled compounds, all variations on olaparib- or rucapariblike structures, are able to cause DNA damage in cancer cells, thereby reducing viability and clonogenic survival and inhibiting tumor growth in subcutaneous or orthotopic xenograft tumors in mice (9*,*^21^–^26^). To date, no clinical trial has been performed to evaluate the safety and efficacy of PARP inhibitor radioligand therapy.

Here, we showed that [^123^I]CC1 binds selectively to PARP, causes damage to DNA double-strand breaks in vitro, and reduces clonogenic survival in vitro and tumor growth in vivo. [^123^I]CC1 also induced increased expression of PARP1 and PARP2 in tumor cells in vitro. Although this may form the basis of a possible feedback mechanism for multiple administrations, as first proposed in Makvandi et al. (26), we did not evaluate this possibility, because a single administration of [^123^I]CC1 (3 MBq) was therapeutically efficacious in mice bearing PSN1 xenografts, despite relatively low tumor uptake (0.9 %IA/g).

Although efficacy in PSN1 cells in vitro was less pronounced than the in vitro response in U87MG cells, in vivo uptake in PSN1 tumors was higher than that in U87MG tumor xenografts, resulting in better therapeutic efficacy. The reason for this contradiction of in vitro versus in vivo was not explored, but possible explanations may include higher uptake of ^123^I in tumor tissue, resulting in a higher absorbed radiation dose; differential radiation sensitivity in vitro versus in vivo; or differences in DNA damage, PARP activation, growth rate, and repopulation in vitro versus in vivo.

We showed that [^123^I]CC1—like [^211^At]MM4 (26) and [^77^Br]RD1 (24*,*^27^) yet unlike [^123^I]MAPi (29), [^123/125^I]KX1 (36), [^125^I]KX-02-019 (37), [^125^I]PARP-01 (26), and [^123^I]GD1 (38)—is effective in reducing tumor growth after a single intravenous administration at relatively low administered activities. In previous work, we proposed that small changes in the structure of PARP inhibitors give rise to major changes in their characteristics (39). Factors affecting the efficacy of radioligand therapy with radiolabeled PARP inhibitors may include PARP binding affinity and selectivity, binding spectrum, bioavailability, pharmacokinetics, and tumor uptake. In addition, trapping of the PARP enzyme by PARP inhibitors, and therefore by radiolabeled PARP inhibitors, may play a significant role in their efficacy. Therefore, it can be expected that different radiolabeled PARP inhibitors may have quite different therapeutic indices (39*,*^40^).

Many studies with radiolabeled PARP inhibitors incorporate Auger electron emitters, because trapping the PARP enzyme brings the PARP inhibitors close to the DNA, an excellent match with the short range of Auger electron emitters. Even though ^125^I is an efficient Auger electron emitter, with some 23 low-energy electrons emitted per decay (5), its long half-life of 60 d may encumber the logistics and radiation safety consideration of radionuclide therapy with this radionuclide. In contrast, ^123^I, with its 14 Auger electrons per decay and a 13.2-h half-life, allows regional distribution from cyclotron production facilities (5).

Normal tissue was minimally affected. The lack of normal-tissue toxicity from [^123^I]CC1 may be explained by the short range of ^123^I’s Auger electrons. Despite high uptake in the gallbladder, intestines, and urinary bladder, because of the luminal uptake of PARP inhibitors in these organs, the impact of the radiation dose from Auger electron emissions will be minimal. In the liver, the uptake of another labeled PARP inhibitor was shown to be cytoplasmic, not nuclear (29). Given that Auger electron emitters decaying in the cytoplasm are 30 times less cytotoxic than those decaying in the nucleus (4), the anticipated cytoplasmic uptake of [^123^I]CC1 in the liver would be far less cytotoxic. The lack of toxicity could not be explained by a lack of interaction between CC1 and murine PARP1, because rucaparib could block uptake of [^123^I]CC1 in normal tissues, such as those of the spleen.

We previously showed that uptake of [^18^F]olaparib in tumor tissue was affected by the administered mass and A_m_ (41). It is likely that this is also the case for [^123^I]CC1 and other PARP inhibitor–based radioligand therapies, although here, we did not assess a range of doses in vivo. Future dose escalation studies are warranted.

The γ-emissions from ^123^I also make it an imaging agent. Therefore, [^123^I]CC1 may be considered a true theragnostic agent, with lower administered doses used for SPECT imaging and larger doses used for therapy. Alternatively, ^18^F-labeled variants (7) or even ^124^I-labeled variants may be used to gauge the relative expression of the target enzyme in tumor tissue during PET imaging.

## CONCLUSION

[^123^I]CC1, an Auger electron–emitting radiopharmaceutical, is promising as a therapeutic strategy for patients with PARP-expressing cancers.

## DISCLOSURE

Bart Cornelissen, Véronique Gouverneur, Thomas Wilson, Zijun Chen, Chung Chan, and Gianluca Destro hold patents on technology relating to the compound in this manuscript. Bart Cornelissen acted as a paid consultant for Theragnostics Ltd. and Blue Earth Diagnostics. No other potential conflict of interest relevant to this article was reported.

## Notes

KEY POINTS **QUESTION:** Can a PARP-targeting radiopharmaceutical radiolabeled with a radionuclide that emits Auger electrons be used for therapy of cancer? **PERTINENT FINDINGS:** We developed [^123^I]CC1—combining the biophysical properties of ^123^I, with its short-range Auger electron emissions, and a small molecule PARP inhibitor—to deliver a dense radiation dose close to cancer cells’ DNA. This allows tumor treatment with minimal damage to healthy surrounding tissue. **IMPLICATIONS FOR PATIENT CARE:** [^123^I]CC1 makes a true theragnostic agent. Given the limited normal-tissue toxicity exerted by [^123^I]CC1, this study could offer a promising therapeutic strategy for patients with PARP-expressing cancers.
